# Risk factors for endogenous endophthalmitis in infectious endocarditis patients

**DOI:** 10.1038/s41433-024-03390-w

**Published:** 2024-10-14

**Authors:** Megh K. Shah, Aretha Zhu, Aditya Uppuluri, Roger K. Henry, Marco A. Zarbin, Neelakshi Bhagat

**Affiliations:** Institute of Ophthalmology & Visual Science, Rutgers New Jersey Medical School, Newark, NJ 07103 USA

**Keywords:** Risk factors, Epidemiology

## Abstract

**Background/Objectives:**

The purpose of this study was to identify demographic variables and systemic comorbidities that may increase risk of endogenous endophthalmitis (EE) development in patients with infective endocarditis (IE).

**Subjects/Methods:**

A retrospective database study was conducted using the 2002–2014 National Inpatient Sample (NIS). Patients with IE and EE were identified using ICD-9-CM codes. Descriptive chi-square and logistic regression analysis identified risk factors for EE in IE patients.

**Results:**

Of 769,472 inpatients with a diagnosis of IE, 2248 had a diagnosis of EE. Women comprised 39.7% of IE patients without EE and 42.6% of those with EE (*p* = 0.005). The majority of IE cases with EE were in those 21–64-year-old (58.5%) age cohort and 67.4% of cases were Whites. Multivariate analysis revealed IE patients in the 21–64 (OR, 3.660) and 65+ age group (OR, 2.852) had increased risk of developing EE compared to the 0–20-year-old group. Hispanic (OR, 1.377) and Asian/Pacific Islander (OR, 1.620) patients had increased risk compared to White patients. Diabetes with (OR, 2.043) and without (OR, 1.433) chronic complications, alcohol use disorder (AUD; OR, 1.795), and cirrhosis (OR, 1.452) conferred an increased risk of developing EE, whereas, congestive heart failure (CHF; OR, 0.716), arrhythmia (OR, 0.678), and having a cardiac device (OR, 0.336) decreased risk of EE in IE subjects.

**Conclusion:**

Older age (21+ years) and Hispanic and Asian/Pacific Islander background were associated with increased risk of developing EE in IE patients. Diabetes with and without chronic complications, AUD, or cirrhosis also conferred a 1.5–2 times increased risk. CHF, arrhythmia, or having a cardiac device were associated with decreased risk.

## Introduction

Endogenous endophthalmitis (EE), a potentially severe purulent infection, results from hematogenous spread of bacteria or fungus from a remote source. It is associated with poor visual outcomes and, in some cases, increased risk of mortality. Approximately, 90% of EE patients have underlying systemic comorbidities such as diabetes and cardiac disease [1, 2]. Infective endocarditis (IE) is one of the most common sources of EE [1, 3–6]. Cases of endophthalmitis resulting from endocarditis with various infective organisms, including culture-negative bacteria (Haemophilus, Aggregatibacter, Cardiobacterium, Eikenella, and Kingella, collectively termed HACEK) and *Candida*, have been documented [7–9]. Previous studies have reported systemic risk factors for EE [1, 2]. In this study, we sought to evaluate the risk factors for EE in patients specifically with IE. Additionally, we also investigated the impact of potential cardiac risk factors, such as history of arrhythmia and valvular disease on developing EE in the setting of IE [10, 11].

A retrospective cross-sectional case-control study was done to identify comorbidities associated with a diagnosis of EE in patients with IE. Identification of risk factors for development of EE in IE patients, can lead to improved vigilance for early diagnosis of a potentially blinding condition.

## Materials/Subjects and methods

The 2002–2014 National Inpatient Sample (NIS) Database was used to perform this retrospective cross-sectional study. The NIS database, developed for the Healthcare Cost and Utilization Project (HCUP), is a publicly available all-payer inpatient healthcare database containing information from about 7 million unweighted hospital admissions nationally each year [12]. The database reports demographic information, including age, sex, and race. Age groups used in this study included <20, 20–64, and >65 year old cohorts, which encompass the paediatric, adult, and older population groups. Previous literature has documented varying rates of IE incidence, risk factor incidence, and mortality between these age groups [13, 14]. The NIS database also includes hospitalization metrics (cost and length of stay, insurance coverage, discharge quarter), current and previous diagnoses, and inpatient procedures. This data is coded in NIS by participating physicians using the International Classification of Diseases, Ninth Revision, Clinical Modification (ICD-9-CM). Weighted data from the NIS database are used to estimate inpatient utilization and outcomes on both a regional and national level [15].

The ICD-9-CM was used in conjunction with the NIS database to identify diagnoses of IE, EE, and systemic comorbidities. First, inpatients with IE were included if they had a diagnosis of bacterial/Candida infectious endocarditis, or a diagnosis of non-specific/non-infectious endocarditis with concurrent septicaemia/Candidemia. Inpatients with concurrent EE were subsequently identified. Cases with concurrent diagnosis of open globe injury were removed to exclude any eyes with traumatic endophthalmitis. A “control” cohort with IE without EE and “case” cohort with both IE and EE were identified. Post-surgical endophthalmitis cases could not be excluded since the ICD-9-CM code for these cases are not reported in the NIS; however, the incidence of post-surgical endophthalmitis reported in literature is very low: 0.02–0.71% [16]. The methodology used in our study to identify EE cases is reported in prior literature [17–19].

ICD-9 diagnosis codes for systemic variables used in this study are reported in Table 1. The study used a validated Johns Hopkins Adjusted Clinical Groups frailty-defining diagnoses indicator algorithm to identify patients with frailty [20]. An algorithm by Tookes et al. [21] was used to identify cases with documented drug use disorder and an IV drug use-associated complication. Column counts in Tables 2 and 3 are based on the total identified cases of EE in the entire isolated cohort of patients with IE. Individual variables may be inconsistently reported for each patient and as such variable totals may not be equivalent to column counts.

**Table 1:** List of variables with corresponding international classification of diseases, ninth revision, codes.

Variable	International classification of diseases, ninth revision, codes
Systemic variables	
Immunosuppression	Composite of V42.0, V42.1, V42.6, V42.7, V42.83, V42.84, 37.51, 33.5–33.52, 33.6, 50.5, 50.51, 50.59, 52.8, 52.80, 52.82, 52.83, 46.97, V87.41, V58.1, V58.11, V58.12, 042, 279.0279.06, 279.09, 279.1–279.13, 279.19, 279.2, 279.3, 279.4, 279.8, 279.9, V58.65, AIDS variable supplied by National Inpatient Sample database
Arrhythmia	427.0, 427.42, 427.31, 427.32, 427.1, 427.9, 427.41
Cardiac device	V45.01, V45.02
Valvular disease	Supplied by National Inpatient Sample database
Organ infection	528.3, 522.4, 521.00, 522.0, 523.10, 522.7, 522.6, 522.5, 461, 462, 463, 464, 465, 481, 482, 491.21, 494, 510, 513, 711.0, 730, 681–683, 686, 590, 597, 599.0, 601, 614, 615, 616, 540–541, 562.01, 562.03, 562.11, 562.13, 556, 567, 5695, 572, 572.1, 575.0, 320, 322, 324, 325, 996.6, 451
Chronic kidney disease	585.1–585.6, 585.9, 586
Cirrhosis	571.2, 571.5, 571.6
Alcohol use disorder	303, 303.0, 303.01, 303.02, 303.03, 303.9, 303.91, 303.92, 303.93, 305.0, 305.01, 305.02, 305.03
Congestive heart failure	Supplied by National Inpatient Sample database
Diabetes without chronic complications	Supplied by National Inpatient Sample database
Diabetes with chronic complications	Composite of variable supplied by National Inpatient Sample database and diabetic retinopathy (362.0, 362.01, 362.02, 362.03, 362.04, 362.05, 362.06)
Diabetic ketoacidosis	250.1–250.13
Hypertension	Supplied by National Inpatient Sample database
Obesity	Supplied by National Inpatient Sample database
IV drug use	Uses algorithm detailed in “Methods”
Radiation therapy	V58.0, V15.3
Tobacco use	305.1, V15.82
Frailty	261, 262, 263.8, 263.9, V77.2, 290.20, 290.21, 290.3, 369.0, 369.00, 369.01, 369.03, 369.04, 369.06, 369.07, 369.08, 707.0, 707.00–707.07, 707.09, 707.20–707.25, 788.34, 788.37, 787.6, V60.0-V60.2, 719.7, 781.2, E880, E880.0, E880.1, E880.9, E884.3

**Table 2:** Descriptive table of demographic variables, hospital stay information.

Variable	No endophthalmitis (*n* = 767224)	Endophthalmitis (*n* = 2248)	*P* value
Gender, no. (%)			**0.005**
Male	462,856 (60.3%)	1292 (57.4%)	
Female	304,228 (39.7%)	957 (42.6%)	
Age group (yrs), no. (%)			
0–20	15,857 (2.1%)	15 (0.7%)	**<0.001**
21–64	379,541 (49.5%)	1314 (58.5%)	**<0.001**
65+	371,780 (48.5%)	919 (40.9%)	**<0.001**
Race, no. (%)			
White	459,297 (70.8%)	1326 (67.4%)	**0.001**
Black	102,274 (15.8%)	260 (13.2%)	**0.002**
Hispanic	52,848 (8.2%)	263 (13.4%)	**<0.001**
Asian or Pacific Islander	12,178 (1.9%)	61 (3.1%)	**<0.001**
Native American	4015 (0.6%)	^a^	**0.039**
Other	17,826 (2.7%)	53 (2.7%)	0.879
Discharge quarter, no. (%)			
1 (January–March)	184,745 (24.1%)	580 (25.8%)	0.064
2 (April–June)	192,449 (25.1%)	495 (22.0%)	**0.001**
3 (July–September)	195,863 (25.6%)	491 (21.8%)	**<0.001**
4 (October–December)	192,929 (25.2%)	683 (30.4%)	**<0.001**
Hospital course			
Average length of stay (days)	13.83	18.03	**<0.001**
Average cost of hospital stay (dollars)	109,191.52	146,501.96	**<0.001**
Death during hospitalization, no. (%)	83,590 (10.9%)	296 (13.2%)	**0.001**
Insurance, no. (%)			
Government	542,979 (70.8%)	1471 (65.4%)	**<0.001**
Private	162,659 (21.2%)	464 (20.6%)	0.509
Other	60,127 (7.8%)	304 (13.5%)	**<0.001**

Bold values represent significant *p* values.

^a^Cells with 1–10 patients are represented with an asterisk in compliance with the Healthcare Cost and Utilization Project guidelines on reporting National Inpatient Sample data.

**Table 3:** Descriptive table of systemic comorbidities.

Systemic comorbidities	No endophthalmitis (*n* = 767,224)	Endophthalmitis (*n* = 2248)	*P* value
Congestive heart failure	143,652 (18.9%)	340 (15.2%)	**<0.001**
Diabetes without chronic complications	151,867 (19.9%)	533 (23.8%)	**<0.001**
Diabetes with chronic complications	59,250 (7.7%)	308 (13.7%)	**<0.001**
Hypertension	378,171 (49.7%)	1083 (48.4%)	0.228
Obesity	57,296 (7.5%)	224 (10.0%)	**<0.001**
Valvular disease	152,994 (20.1%)	572 (25.5%)	**<0.001**
Immunosuppression	21,079 (2.7%)	46 (2.0%)	**0.042**
Arrhythmia	216,562 (28.2%)	456 (20.3%)	**<0.001**
Cardiac device	44,216 (5.8%)	36 (1.6%)	**<0.001**
Organ infection	128,521 (16.8%)	377 (16.8%)	0.998
Chronic kidney disease	157,938 (20.6%)	467 (20.8%)	0.825
Cirrhosis	19,680 (2.6%)	115 (5.1%)	**<0.001**
Alcohol use disorder	9448 (1.2%)	64 (2.8%)	**<0.001**
Diabetic ketoacidosis	4085 (0.5%)	39 (1.7%)	**<0.001**
Radiation therapy	1556 (0.2%)	^a^	0.811^b^
Tobacco use	97,600 (12.7%)	230 (10.2%)	**<0.001**
IV drug use	60,597 (7.9%)	212 (9.4%)	**0.007**
Frailty	114,594 (14.9%)	440 (19.6%)	**<0.001**

Bold values represent significant *p* values.

^a^Cells with 1–10 patients are represented with an asterisk in compliance with the Healthcare Cost and Utilization Project guidelines on reporting National Inpatient Sample data.

^b^*P* values denoted by two asterisks indicate Fisher’s exact tests were used.

Statistical analysis was performed with IBM SPSS software v25 (IBM, Armonk, NY). Chi square testing compared demographics, systemic comorbidities, morbidity during hospitalization, and discharge quarter between case and control cohorts. Independent samples t-testing was used to compare cost per day and length of stay. Binary logistic regression analysis was performed with IBM SPSS v25 to identify risk factors for EE in cases with IE. Univariate regression used the level of significance *p* < 0.05. Bonferroni correction was applied to the multivariate logistic regression tests to minimize the type I error.

This study, which adhered to the tenets of the Declaration of Helsinki, received exemption by the Rutgers University Institutional Review Board due to non-human determination according to the National Bureau of Economic Research [22].

## Results

Of 769,472 total IE patients included in the study, 2,248 (0.29%) had EE. Mean ages of cases (IE patients with EE) and controls (IE patients without EE) were 59.5 years and 61.4 years (*p* < 0.001), respectively. Table 2 shows results of chi square analysis and independent samples t-testing comparing the percentage of IE patients with and without EE across demographic variables (gender, age group, race) and hospital stay information (discharge quarter, hospital course, insurance). Women comprised 42.6% of cases but only 39.7% of controls (*p* = 0.005). Most IE patients with and without EE were White. Descriptive statistical analysis (Table 2) noted a significantly higher proportion of cases (with endophthalmitis) than controls in the 21–64 aged cohort, 58.5% vs 49.5% (*p* < 0.001), respectively. The average length of stay was significantly longer in IE patients with EE (18.03 days vs. 13.8 days; *p* < 0.001). The average cost per day of hospitalization was also significantly higher in IE patients with EE ($146,501.96) than those without ($109,191.52; *p* < 0.001). The mortality rate for EE group was 13.2% compared to 10.9% in the non-EE group (*p* = 0.001).

Table 3 presents the results of chi-square analysis comparing the prevalence of systemic comorbidities. Diabetes with and without chronic complications, obesity, valvular disease, liver cirrhosis, alcohol use disorder, diabetic ketoacidosis, IV drug use, and frailty were significantly more prevalent in IE patients with EE (*p* < 0.05). Meanwhile, congestive heart failure, immunosuppression, arrhythmia, history of cardiac device, and tobacco use were less prevalent in IE patients with EE (*p* < 0.05).

Univariate and multivariate logistic regression analysis of demographic variables and systemic conditions associated with a significantly increased or decreased risk of EE are presented in Table 4. There was no significant gender difference in risk of developing EE. With respect to age, IE patients in the 21–64 (odds ratio [OR], 3.660; *p* < 0.001) and 65+ (OR, 2.852; *p* = 0.001) year old cohorts showed a significantly increased risk of developing EE compared to patients in the 0–20-year-old age group. With respect to race, Hispanic (OR, 1.377; *p* < 0.001) and Asian/Pacific Islander (OR 1.620; *p* < 0.001) patients had a greater risk of EE compared to White patients. Among systemic comorbidities, diabetes without chronic complications (OR, 1.433; *p* < 0.001), diabetes with chronic complications (OR, 2.043; *p* < 0.001), cardiac valvular disease (OR, 1.422; *p* < 0.001), cirrhosis (OR, 1.452; *p* = 0.001), and alcohol use disorder (OR, 1.795; *p* < 0.001) were associated with an increased risk of developing EE. Meanwhile, congestive heart failure (OR, 0.716; *p* < 0.001), arrhythmia (OR, 0.678; *p* < 0.001), and history of cardiac device (OR, 0.336; *p* < 0.001) were associated with a decreased risk. Our results did not show a statistically significant difference in developing EE with obesity, immunosuppression, diabetic ketoacidosis, tobacco use, IV drug use (IVDU), and frailty using multivariate analysis.

**Table 4:** Results of logistic regression.

Variable	Univariate	*P* value	Multivariate	*P* value
Gender				
Male	Reference	–	Reference	–
Female	1.127 (1.036–1.225)	0.005	1.090 (0.987–1.204)	0.089
Age group (yrs)				
0–20	Reference	–	Reference	–
21–64	3.616 (2.180–5.998)	**<0.001**	3.660 (1.965–6.818)	**<0.001**
65+	2.580 (1.554–4.285)	**<0.001**	2.852 (1.527–5.324)	**0.001**
Race				
White	Reference	–	Reference	–
Black	0.881 (0.771–1.006)	0.062	–	–
Hispanic	1.725 (1.510–1.969)	**<0.001**	1.377 (1.200–1.580)	**<0.001**
Asian or Pacific Islander	1.744 (1.349–2.254)	**<0.001**	1.620 (1.252–2.096)	**<0.001**
Native American	0.398 (0.159–0.993)	0.048	0.328 (0.131–0.819)	0.017
Other	1.028 (0.781–1.354)	0.842	–	–
Systemic comorbidities				
Congestive heart failure	0.771 (0.687–0.865)	**<0.001**	0.716 (0.622–0.824)	**<0.001**
Diabetes without chronic complications	1.253 (1.137–1.381)	**<0.001**	1.433 (1.270–1.616)	**<0.001**
Diabetes with chronic complications	1.898 (1.683–2.141)	**<0.001**	2.043 (1.763–2.368)	**<0.001**
Hypertension	0.950(0.874–1.032)	0.224	–	–
Obesity	1.369 (1.192–1.571)	**<0.001**	1.014 (0.855–1.203)	0.869
Valvular disease	1.364 (1.240–1.500)	**<0.001**	1.422 (1.268–1.593)	**<0.001**
Immunosuppression	0.741 (0.553–0.992)	0.044	0.865 (0.621–1.204)	0.390
Arrhythmia	0.647 (0.584–0.718)	**<0.001**	0.678 (0.599–0.769)	**<0.001**
Cardiac device	0.267 (0.192–0.371)	**<0.001**	0.336 (0.235–0.479)	**<0.001**
Organ infection	1.000 (0.895–1.117)	0.999	–	–
Chronic kidney disease	1.011 (0.913–1.120)	0.829	–	–
Cirrhosis	2.053 (1.701–2.478)	**<0.001**	1.452 (1.156–1.825)	**0.001**
Alcohol use disorder	2.357 (1.837–3.023)	**<0.001**	1.795 (1.313–2.455)	**<0.001**
Diabetic ketoacidosis	3.269 (2.375–4.499)	**<0.001**	1.687 (1.116–2.549)	0.013
Radiation therapy	1.097 (0.456–2.642)	0.836	–	–
Tobacco use	0.783 (0.683–0.898)	**<0.001**	0.811 (0.693–0.948)	0.008
IV drug use	1.212 (1.052–1.397)	0.008	1.068 (0.892–1.279)	0.473
Frailty	1.385 (1.248–1.538)	**<0.001**	1.113 (0.977–1.267)	0.106

Bold values represent significant *p* values.

Bonferroni correction: new alpha cutoff = **0.0029** (0.05/17). Bold value represents the new alpha cutoff after Bonferroni correction, which was applied to this Table.

## Discussion

In our population of IE patients, 0.29% had EE. While there is a dearth of literature on the prevalence of EE in patients with IE, risk factors for development of EE as reported in the literature include: IVDU, bacteraemia, endocarditis, urinary tract infections, dental abscess, liver abscess, COVID-19 infection, indwelling central venous catheters, presence of a pacemaker, contaminated IV infusion, and procedures such as endoscopy [19, 23–32]. While previous literature has reported IE as a risk factor for EE [33, 34], this study aims to explore risk factors for EE in patients admitted to hospitals with IE.

Systemic comorbidities that increased the risk of developing EE in IE patients were diabetes with (2-fold) and without (1.4-fold) chronic complications, cardiac valvular disease (1.4-fold), cirrhosis (1.5-fold), and alcohol use disorder (1.8-fold). Individuals with diabetes exhibit poor phagocytic function and cellular immunity which can contribute to an increased risk of infections and infectious complications [35–37]. Diabetes-induced endothelial dysfunction may promote the risk of bacterial colonization and infection [36, 38]. Another condition associated with an increased risk of the development of EE in IE patients was cardiac valvular disease. Cardiac valvular disease can increase risk of vegetations, as injured endothelium can cause microthrombosis [39]. These vegetations are shielded from access by the host immune system and as such can become colonized by bloodborne bacteria that can cause microvascular embolization like EE [39]. Pre-existing cardiac valvular disease undergoing surgical procedures such as valve replacement may further increase the risk of septicaemia and EE [40]. Further, postsurgical or postprocedural infections may also contribute to the development of EE in patients with cardiac valvular disease [41, 42].

While IVDU is a well-established risk factor for the development of endocarditis, our study did not find that it increased the risk of EE in IE patients [19, 32]. In the current study, alcohol use disorder showed a significantly increased risk of EE development in IE patients. Alcohol use disorder can result in immunomodulation through derangements of the hypothalamic-pituitary-adrenal axis, the cytokine profile, and immune cell function [43–45]. Cirrhosis has been shown by the current study and prior studies to be a risk factor for the EE [46–48]. Cirrhosis can disrupt the mounting of normal immune responses and can cause cirrhosis-associated immunodeficiency, a factor that may promote a more severe infection [49]. Decreased circulating albumin in cirrhosis causes a lack of sequestration of PGE_2_, a molecule that normally promotes immunosuppression [49].

Older age was another risk factor for the diagnosis EE in our cohort of IE. Multivariate analysis showed that the two older age cohorts, 21–64 and 65+ years, were significantly more likely to develop EE compared to the 0–20 year group. Aging is associated with the development of physiological immunosenescence, a process that has been linked to an increase in the risk for bacterial infections, diabetes, and malignancies in older individuals, which may explain the higher odds of developing EE with age [50]. Frailty was found to be a significant risk factor for EE on univariate analysis, but not on multivariate analysis. In a 2022 retrospective study of septicaemic patients using the NIS database from the same time period as the current study, Henry et al. found frailty to be a significant risk factor for EE in older patients with septicemia [20]. Many of the frailty defining diagnoses have been associated with factors that may predispose patients to EE [20]. For example, cognitive decline and poor nutrition status have been associated with retinal microvascular abnormalities in the elderly [20].

Interestingly, the current study found a decrease in the risk of EE in IE patients with arrhythmias, cardiac devices, or congestive heart failure. The risk of endophthalmitis in IE patients with heart failure and cardiac devices has not been well described in the literature, but many case reports of infected cardiac devices resulting in endophthalmitis are published [30, 42, 51–53]. As per our communication with cardiologists, this seemingly inverse relationship could be explained by the fact that these cardiac patients may routinely undergo annual or biannual echocardiography as part of their care, which may identify endocarditis earlier in the clinical course, starting antibiotics earlier in the course decreasing the risk of sepsis and septic emboli [54]. It is uncertain why certain cardiac risk factors in our study, e.g., valvular disease, were associated with an increased risk of EE, while others, e.g., arrhythmia, were not. In a study by Uppuluri et al. on patients with atrial fibrillation and atrial flutter, endocarditis was associated with significantly increased risk of central retinal artery occlusion (CRAO) on univariate analysis [55]. The mechanism of CRAO in these patients is emboli to the retinal arterial system from vegetations in IE. These septic emboli have the potential to seed the affected eye with pathogens and cause endophthalmitis as well, although the current study showed a decreased risk of developing EE in those IE subjects with arrhythmias. Further study is needed to elucidate the relationship between arrhythmias and EE in IE patients. Although our study did not investigate prevalence of comorbid CRAO, consideration should be given to risk of developing CRAO and EE in IE patients, as risk factors for EE such as valvular disease and diabetes are risk factors for CRAO as well [55–57].

It is also interesting to note that while systemic factors that can cause secondary immunosuppression (e.g., diabetes, cirrhosis, and older age) were risk factors for EE in our study, primary immunosuppression was not. This may be due to diligent vigilance to diagnose infection as early as possible in primary immunosuppression (patients with organ transplantation, or patients undergoing cancer treatment etc). The goal is to start recognize and treat infection before septicaemia occurs to prevent septic emboli from reaching various organs like eyes; whereas in chronic diseases with secondary immunosuppression, smouldering infections can be recognized late increasing the risk of septic emboli to cause IE. In addition, when considering comorbidities such as diabetes and cirrhosis, other mechanisms in addition to immunosuppression may increase risk of EE, such as endothelial dysfunction in diabetic patients, and translocation of enteric bacteria in cirrhosis patients [36, 58, 59].

Hispanic and Asian or Pacific Islander patients with IE had a significantly increased risk of developing EE when compared to White patients in this study. A 2021 retrospective study by Seidelman et al. [60] on 771 patients with *Candidemia* showed that Non-Caucasian/White individuals had significantly greater odds (1.65-fold) of developing Candida endophthalmitis compared to Caucasian/White individuals. Further studies are needed to elucidate how such racial differences are attributable to genetic or socioeconomic factors. Socioeconomic factors, including insurance status and education level, can also influence management of risk factors that may contribute to an increased risk of EE as well as management and treatment of EE [61].

Patients with EE secondary to IE had significantly greater average cost during the hospital stay, increased length of hospital stay, and increased death rate during hospitalization. These findings are likely due to a more severe and disseminated disease course in patients with EE requiring more intense management. As EE can often be the first manifestation of IE, a high index of suspicion may allow for prompt diagnosis and identification of the source of infection [29]. Further research is needed on the utility of screening for IE in patients with EE, as one negative TEE examination cannot be used to rule out endocarditis since patients may become positive later on in the disease course [9, 34]. The current results may support increased consideration of IE in patients with EE. Additionally, the prompt identification and treatment of EE secondary to IE may impact a patient’s clinical course. EE may be categorized by the infectious agent that is causing the endophthalmitis. Most cases of endogenous bacterial endocarditis (EBE) present acutely with 90% of patients reporting decreased vision and 50% having eye pain [33]. EBE generally has poor visual outcomes [33]. While data are minimal regarding the utility of screening for EE in patients with IE, one study on 111 patients meeting Duke criteria for endocarditis found that those patients who reported no visual symptoms had no retinal changes in need of intervention on dilated fundus examination, while those with symptoms had complications such as subretinal abscess [62]. As such, patients with a diagnosis of bacterial endocarditis, especially those with vision symptoms or risk factors such as diabetes, alcohol use, or cirrhosis may require a higher index of suspicion for EE although no screening guidelines currently exist. Further study is warranted on this topic. Conversely, endogenous fungal endophthalmitis (EFE) generally presents with an indolent course, with *Candidemia* being the most common cause [33]. EFE generally begins as chorioretinitis and progresses over time to vitritis and endophthalmitis [18, 33]. While the slowly progressive nature of endogenous fungal endophthalmitis seems to lend itself to early diagnosis and intervention, routine ophthalmologic screening after identification of *Candida* septicaemia has not been supported by the American Academy of Ophthalmology [18, 63].

### Limitations

The National Inpatient Sample is the largest publicly available all-payer inpatient healthcare database [12]. As such, the first limitation of this study is that the cases were only inpatients, as outpatients with IE were not included. Additionally, the diagnosis of endophthalmitis was made during the patient’s inpatient stay. As with any discharge database, causality cannot be determined as NIS does not provide a temporal relationship between the development of endophthalmitis, endocarditis, and the risk factors under interrogation. Additionally, the NIS does not contain detailed information about the patients with endophthalmitis including laterality of ocular involvement or presence of bilateral disease, visual acuity, duration of illness, and treatments provided. The use of ICD-9 codes does not allow our analysis to expand beyond those risk factors and diagnoses that have codes. As such, the codes used in this analysis may not be all encompassing. Methodology to identify cases used here has been described in the literature [17–19, 28]. Finally, physicians may report codes inconsistently for patients or report data inaccurately, which may introduce error, as the quality of the provided data is dependent on the discharges from hospitals participating in the HCUP. Despite these limitations, we believe that this analysis using a nationally representative database represents an important contribution to the extant literature.

This study identified risk factors for the development of EE in a cohort of inpatients with IE. Age >20 years was associated with increased odds of developing concurrent EE. Hispanic and Asian or Pacific Islander patients had higher odds of developing EE as compared to Whites. There was no increased risk of EE with respect to the gender. The presence of diabetes with or without chronic complications, cardiac valvular disease, liver cirrhosis, and alcohol use disorder were each significantly associated with the presence of EE. The presence of congestive heart failure, arrhythmia, or a cardiac device significantly decreased the odds of developing EE. Patients with endophthalmitis had significantly greater inpatient lengths of stay, greater average cost of the hospitalization, and greater mortality. Being aware of the characteristics of these high-risk patient groups can increase physician awareness of infectious seeding in the eye and can raise the index of suspicion of EE when managing patients with IE. This heightened awareness can aide in early diagnosis, screening, and treatment of EE, which can have devastating visual complications if not treated promptly.

## Summary

### What was known before

- Infectious endocarditis (IE) is a risk factor for endogenous endophthalmitis (EE), but the risk factors for EE in IE patients were not well understood.
- Previous studies have shown that certain demographic variables and comorbidities may influence the risk of EE in other infections.

### What this study adds

- This study identified several risk factors for EE in IE patients, including age, ethnicity, and specific comorbidities.
- Older IE patients (21–64 years and 65+ years) and those of Hispanic or Asian/Pacific Islander ethnicity had a higher risk of developing EE.
- Comorbidities such as diabetes, alcohol use disorder, and cirrhosis increased risk of EE, while congestive heart failure, arrhythmia, and having a cardiac device decreased risk.

## Data Availability

The datasets generated during and/or analysed during the current study are available in the 2002–2014 HCUP National Inpatient Sample (NIS) Database, www.hcup-us.ahrq.gov/databases.jsp.
